# Reduced Prefrontal Cortex Hemodynamic Response in Adults with Methamphetamine Induced Psychosis: Relevance for Impulsivity

**DOI:** 10.1371/journal.pone.0152373

**Published:** 2016-04-06

**Authors:** Kazuhiko Yamamuro, Sohei Kimoto, Junzo Iida, Naoko Kishimoto, Yoko Nakanishi, Shohei Tanaka, Toyosaku Ota, Manabu Makinodan, Toshifumi Kishimoto

**Affiliations:** 1 Department of Psychiatry, Nara Medical University School of Medicine, Kashihara, Japan; 2 Faculty of Nursing, Nara Medical University School of Medicine, Kashihara, Japan; Chiba University Center for Forensic Mental Health, JAPAN

## Abstract

Patients with methamphetamine abuse/dependence often exhibit high levels of impulsivity, which may be associated with the structural abnormalities and functional hypoactivities observed in the frontal cortex of these subjects. Although near-infrared spectroscopy (NIRS) is a simple and non-invasive method for characterizing the clinical features of various psychiatric illnesses, few studies have used NIRS to directly investigate the association between prefrontal cortical activity and inhibitory control in patients with methamphetamine-induced psychosis (MAP). Using a 24-channel NIRS system, we compared hemodynamic responses during the Stroop color-word task in 14 patients with MAP and 21 healthy controls matched for age, sex and premorbid IQ. In addition, we used the Barrett Impulsivity Scale-11 (BIS-11) to assess impulsivity between subject groups. The MAP group exhibited significantly less activation in the anterior and frontopolar prefrontal cortex accompanied by lower Stroop color-word task performance, compared with controls. Moreover, BIS-11 scores were significantly higher in the MAP group, and were negatively correlated with the hemodynamic responses in prefrontal cortex. Our data suggest that reduced hemodynamic responses in the prefrontal cortex might reflect higher levels of impulsivity in patients with MAP, providing new insights into disrupted inhibitory control observed in MAP.

## Introduction

Methamphetamine (MA) abuse/dependence is a global public health problem. Approximately 5% of the adult population of the United States has used MA at least once, and there are ~35 million MA users worldwide including Japan [1, 2]. MA abuse can cause persistent psychosis that is characterized by positive and negative symptoms similar to those observed in people with schizophrenia. Such outcomes are common, and may occur in individuals with no history of psychiatric illness [3, 4]. This disease process is termed methamphetamine induced psychosis (MAP), and is often associated with high levels of psychiatric hospitalization and serious social dysfunction [5]. Indeed, individuals who repeatedly use MA or who self-administer high doses commonly develop MAP [6, 7]. MAP may be transient, and recovery may occur within 1 month after discontinuing MA use [8–10]. However, 30% of MAP sometimes prolong and often persists despite long periods of MA abstinence for up to 6 months [11], and 10–28% of MAP had symptoms persist more than 6 months [8, 11]. Despite frequent poor outcomes, little is known about the pathophysiology and the effective treatment strategy for MAP.

Inhibitory control can be defined as the ability to regulate or inhibit prepotent attentional or behavioral actions. A deficit in the ability to inhibit prepotent responses is associated with impulsivity [12, 13]. Indeed, neuropsychological examinations have revealed disrupted inhibitory control in patients with MA abuse/dependence [14, 15], which persists during abstinence from MA [16, 17]. Furthermore, MA abusers frequently exhibit high impulsivity, which is associated with higher rates of aggression and high-risk behavior [18, 19]. Collectively, these data suggest that MA abuse/dependence might lead to deficits in the behavioral inhibition system [20].

Inhibitory control can be clinically assessed using the go/no-go and stop-signal tasks as well as the Stroop color-word task. Indeed, regional brain activities elicited during the Stroop color-word task have been measured using functional magnetic resonance imaging (fMRI) for the assessment of cognitive control [21–23]. Previous fMRI studies have reported that MA abusers exhibit lower activity in the prefrontal cortex (PFC) or other regions during cognitive tasks, including the Stroop-color word task [24–26]. Near-infrared spectroscopy (NIRS) has been recently established as a simple and noninvasive bedside technique for monitoring hemodynamic changes during cognitive task performance. When measuring changes in oxygenated hemoglobin ([oxy-Hb]) and deoxygenated hemoglobin ([deoxy-Hb]) concentrations, [oxy-Hb] changes during brain activation are thought to represent alterations in regional cerebral blood volume [27, 28]. As NIRS is relatively insensitive to motion artifact and has a high temporal resolution, it is considered to be a reliable tool for monitoring the time course of prefrontal activity during cognitive performance [29, 30]. Therefore, NIRS has increasingly been used to evaluate individuals with psychiatric disorders which are at least in part characterized by high impulsivity [31–34]. However, no previous NIRS studies have directly examined the relationship between MAP and hemodynamic responses during the Stroop color-word task.

In the present study, we used NIRS to examine prefrontal hemodynamic responses during the Stroop color-word task in patients with MAP. We hypothesized that patients with MAP would show a reduced prefrontal hemodynamic response during the cognitive task. Given that prefrontal cortical activation measured by fMRI was reported to correlate with the Barret Impulsiveness Scale (BIS)-measured impulsivity [35–38], the size of the hemodynamic response might correlate with the degree of impulsivity as measured by Barret Impulsiveness Scale, Version 11 (BIS-11). To test this, we used a multi-channel NIRS method to examine changes in prefrontal cerebral blood volume during the Stroop color-word task in patients with MAP and controls.

## Material and Methods

### Participants

Participants were recruited from September, 2014 to August, 2015. We recruited 14 MAP patients (9 males: mean ± SD age 42.6 ± 11.6 years; and 5 females: mean ± SD age 34.4 ± 9.6 years) by consecutive sampling, from the outpatient clinic in the Department of Psychiatry at Nara Medical University, Japan (Table 1). At least two experienced psychiatrists reached a consensus regarding DSM-IV-TR and ICD-10 diagnoses for each participant via structured interviews and reviews of medical records. Exclusion criteria included neurological disorders, a history of traumatic brain injury, electroconvulsive therapy, serious medical conditions, low premorbid IQ (below 70), any other substances dependence and psychiatric comorbidity. Since the patients with MAP were treated with typical (n = 5) or atypical (n = 7) antipsychotics, anticholinergics (n = 6) and/or anxiolytics (n = 12), we assessed the chlorpromazine-, biperiden- and diazepam-equivalent doses as described previously [39, 40]. For healthy controls, 21 control subjects (16 males: mean ± SD age 41.9 ± 10.1 years; and 5 females: mean ± SD age 43.6 ± 7.8 years) were recruited through local print advertising in this study (Table 1). The absence of a psychiatric diagnosis was confirmed using a standard clinical assessment that included a psychiatric evaluation and Structured Clinical Interview for DSM-IV Axis Disorders Non-Patient Edition (SCID-NP). All participants were of Japanese descent and right-handed, as indicated by the Edinburgh Handedness Inventory [41]. Those only are admitted who have completed the middle school course or gone through a similar course of study. A trained psychologist assessed the premorbid IQs of each subject by using the Japanese version of the National Adult Reading Test (JART) [42]. All psychological assessments were performed along with NIRS exam on the same day.

**Table 1:** Participant characteristics.

Variable	Patients with MAP		Controls		*p* value
	(n = 14)		(n = 21)		
	Mean	SD	Mean	SD	
Age (years) ^a^	39.64	11.20	42.16	8.30	0.45
Male/female^b^	9/5		16/5		0.47
JART IQ ^a^	93.67	12.17	101.68	7.82	0.21
Age at first use of methamphetamine	21.71	5.59	NA		NA
Age of onset of psychotic symptoms	33.00	12.11	NA		NA
Duration of illness (years)	7.84	4.73	NA		NA
Chlorpromazine equivalent dose (mg/day)	467.81	369.06	NA		NA
Biperiden equivalent dose (mg/day)	0.64	0.99	NA		NA
Diazepam equivalent dose (mg/day)	27.79	21.43	NA		NA
Number of hospitalizations	2.86	3.66	NA		NA
Duration of hospitalizations (months)	4.85	6.62	NA		NA

MAP, methamphetamine induced psychosis; JART, Japanese Adult Rating Test; SD, standard deviation; NA, not applicable

^a.^ Student’s *t*-tests were used

^b.^ *χ*^2^ test was used

The study protocol was approved by the appropriate Ethics Committees at Nara Medical University and was in accordance with the Declaration of Helsinki. All study participants or their legal guardians provided written informed consent for their participation prior to the start of the study.

### Psychopathological evaluation

#### Positive and Negative Symptom Scale (PANSS)

Using the Japanese version of the Positive and Negative Symptom Scale (PANSS) [43], members of the certificated psychiatrists (KY, SK, TO and MM) evaluated clinical symptoms in the participants with MAP. Specifically, we used a seven point Likert-scale, in which higher scores indicate greater severity, to rate all of the items. Subscale scores were calculated using small sets of variables based on the three domains of the PANSS: positive, negative, and general psychopathological symptoms.

#### Barratt Impulsiveness Scale, Version 11 (BIS-11)

The current BIS-11 contains a 30-item checklist for the measurement of impulsivity [44]. Participants indicate whether phrases describing aspects of impulsivity pertain to themselves using a scale ranging from “1” to “4”: 1) rarely/never, 2) occasionally, 3) often, and 4) almost always. The BIS-11 can be divided into 3 subscales that assess different aspects of impulsivity. The motor impulsivity subscale concerns spontaneous motor actions and includes statement such as, “I do things without thinking”. The attentional impulsivity subscale assesses one’s ability to maintain focused attention on a task and includes statements such as “I concentrate easily”. The non-planning impulsivity subscale pertains to a lack of concern for the future and includes statements such as, “I plan tasks carefully”. Numerous recent studies have indicated that the BIS-11 and its subscales are reliable self-report instruments for measuring impulsivity [44, 45].

### The Stroop color-word task

Using methods similar to those described previously [46], we asked all participants to complete the Stroop color-word task. In general, the Stroop color-word task consists of two pages. In our experiment, the items on the first page included the color-words RED, GREEN, and BLUE printed in black ink. The items on the second page included the words RED, GREEN, and BLUE printed in red, green, or blue ink, with the limitation that the word meaning and ink color could not match. The items were randomly distributed on the two pages, with the exception that no item within a column could follow itself.

Before starting the task, a well-trained psychologist informed the participants of the following instructions: “This is a test of how quickly you can read the words on the first page, and say the colors of the words on the second page. After you have read the words on the first page for 45 s, we will turn the page. Then you will be required to say the colors of the words on the second page for 45 s. We will repeat this process with the second page three times. Then we will show you the first page again.”

**Fig 1:**
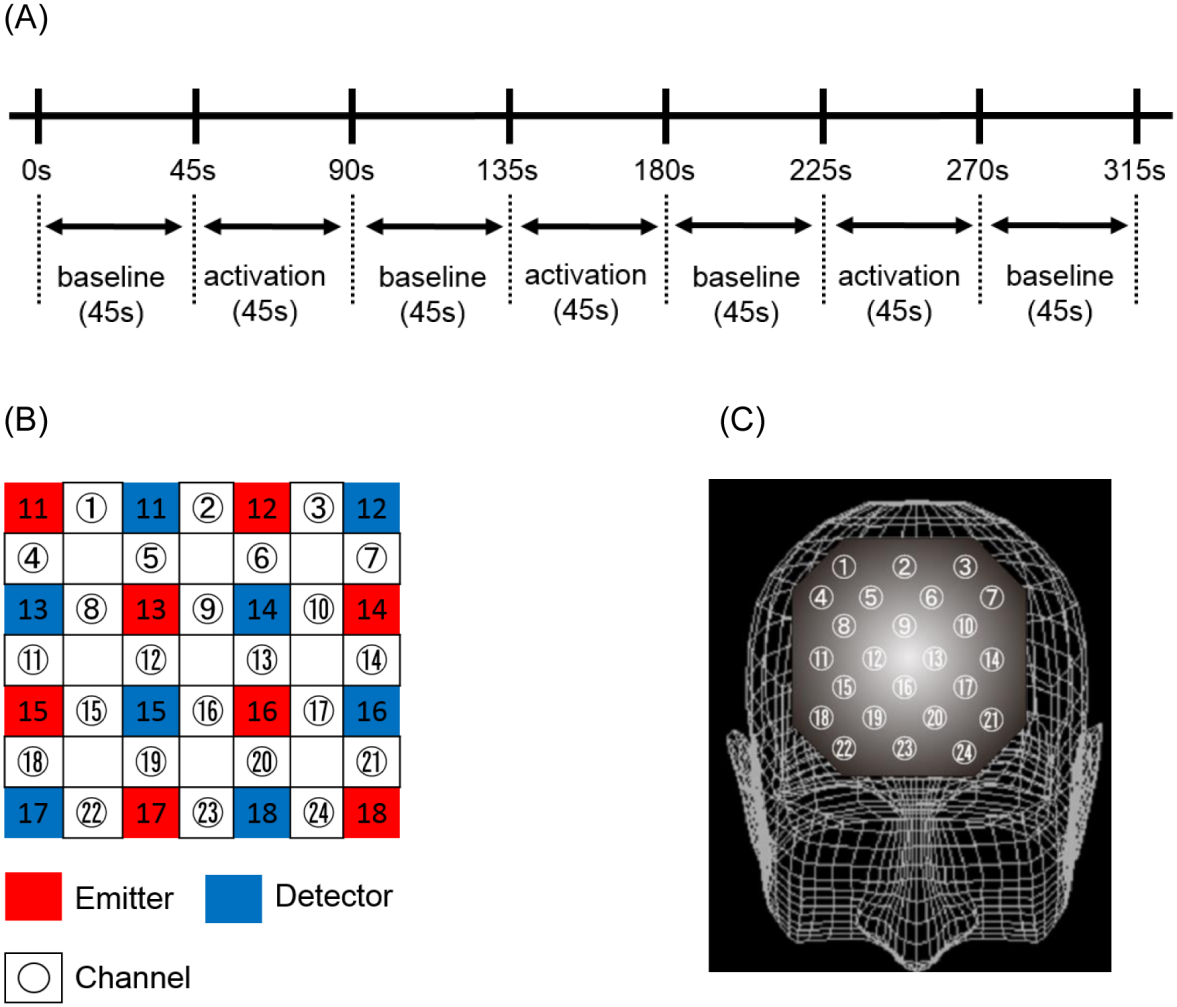
Location of the 24 channels in the near-infrared spectroscopy instrument. (A) Timeline of stimulus presentations. The baseline Stroop color-word task was presented, the activation Stroop color-word task was repeated three times, and then the baseline task was presented again. Each task took 45 s. (B) Arrangement of emitters and detectors according to the definition of each channel. (C) Corresponding anatomical site of each channel.

We used a design for the Stroop color-word task that has been found to be simpler and easier, as described previously [47, 48]. Briefly, the entire sequence of the Stroop color-word task consisted of a 45-s task (p1), a 45-s task (p2) repeated three times, and then the p1 task for 45-s again (Fig 1(a)). The 45-s, p1 task was designated as the baseline task. We counted the number of correct answers for each task and also evaluated the task performance. The number of correct answers for each trial on the Stroop color-word task was identified using the following terms: Stroop color-word task number of correct answers for the first presentation (SCWC-1), Stroop color-word task number of correct answers for the second presentation (SCWC-2) and Stroop color-word task number of correct answers for the third presentation (SCWC-3), as described previously [47]. Meanwhile, we also evaluated the mean percentage of incorrect answers on a series of presentations on the Stroop color-word task. The design—a word-reading task (baseline task) and an incongruent color-naming task (activation task)–was intended to be as easy as possible while meeting NIRS study requirements [49], thus allowing a direct comparison of results between studies [31, 47, 48, 50].

### NIRS measurements

**Fig 2:**
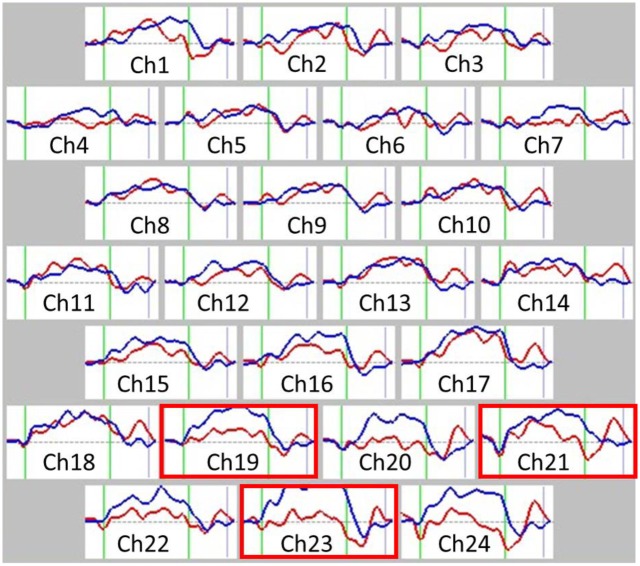
Grand average waveforms showing changes in oxyhemoglobin during the Stroop color-word task. Near-infrared spectroscopy (NIRS) waveforms (changes in oxy-Hb concentration) between methamphetamine induced psychosis (MAP) and control groups recorded during the Stroop color-word task in 24 channels. Red lines represent the MAP group, while blue lines represent the control group. Gray dotted lines represent baseline [oxy-Hb]. The task was performed in the interval represented by green lines; the first green line indicates the beginning of the task and the second indicates the end. Gray lines represent the time points when [oxy-Hb] waveforms return to the post-task baseline. Statistically significant regions are shown via red frames (Ch 19, 21, and 23). Group differences were tested using a multiple comparison test with a false discovery rate (FDR) correction (*p*<0.05). Abbreviations: Ch, channel.

NIRS can detect changes in the concentration of cerebral blood hemoglobins, such as [oxy-Hb] and [deoxy-Hb]. The principle of NIRS is based on the modified Lambert-Beer law, and NIRS monitors the absorption of near-infrared light by [oxy-Hb] and [deoxy-Hb] using two different wavelengths. Increasing [oxy-Hb] and decreasing [deoxy-Hb], as measured by NIRS, have been shown to reflect cortical activation [51, 52]. Rodent studies have indicated that, of the two measures, [oxy-Hb] is a more sensitive indicator of regional cerebral blood flow, because the direction of change of [deoxy-Hb] is determined by the degree of change in venous blood oxygenation and volume [53]. Therefore, we decided to focus on changes in [oxy-Hb]. We measured [oxy-Hb] using a 24-channel NIRS machine (Hitachi ETG-4000, Hitachi Medical Corporation, Tokyo, Japan), which determined the absorption of two wavelengths of near infrared light (760 and 840 nm). [Oxy-Hb] was calculated as previously described [54]. The inter-probe intervals of the machine were set at 3.0 cm, and it was determined that the machine measured points 2–3 cm beneath the scalp, which corresponds with the depth of the surface of the cerebral cortices [52]. Participants maintained a natural sitting position during NIRS measurements. The NIRS probes were placed over the prefrontal regions of each participant, and arranged to measure relative [oxy-Hb] concentration changes at 24 measurement points in an 8- × 8-cm area (Fig 1(b)). The midcolumn of the probe were located over Fpz with the lowest probes located along the Fp1-Fp2 line, in accordance with the international 10–20 system used in electroencephalography. The probe positions and measurement points over the cerebral cortex were confirmed by overlaying the probe positions on a three-dimensionally reconstructed MRI scan of the cerebral cortex of a representative participant from the control group (Fig 1(c)). Cortical regions were estimated for each channel using a virtual registration method [55]. The probe arrangement allowed us to measure [oxy-Hb] concentration changes in the following cortical surface regions: the frontopolar and anterior PFC (FPPFC and APFC; Brodmann area (BA) 10, dorsolateral PFC (DLPFC; BA 9 and 46) and ventrolateral PFC (VLPFC; BA44 and 45). The absorption of near-infrared light was measured with a time resolution of 0.1 s, and data were analyzed using the “integral mode”. The pre-task baseline for each participant comprised the mean activity during the 10 s immediately preceding the task, and the post-task baseline encompassed the mean activity during the 25 s immediately following the task. Linear fitting was performed on the data between the two baselines [31, 47]. Moving average methods (moving average window, 5 s) were used to exclude short-term motion artifacts in the analyzed data. We attempted to minimize motion artifacts by closely monitoring participants, and by instructing them to avoid artifact-evoking body movements, such as neck movements, strong biting, and blinking. We particularly sought to reduce the frequency of blinking because it was identified as the most influential source of motion artifacts in a preliminary artifact-evoking study. Examiners who were blind to the diagnoses of the participants analyzed the NIRS results. Hence, we referred to the relative [oxy-Hb] as the difference in [oxy-Hb] between baseline and activation tasks (Fig 2): the time course of [oxy-Hb] changes were assessed during the period from the beginning of activation task until the point when [oxy-Hb] waveform returned to the baseline, and these values were averaged from three repeated trials to reduce the influence of potential accidental changes and participant fatigue, similar to the method described previously [31, 47, 50].

### Statistical Analyses

We used *χ*^2^ test to examine the group difference for categorical variable (i.e., male and female) In addition, the clinical variables that fit the normal distribution were compared using Student’s *t*-tests while the clinical variables not normally distributed were analyzed using the Mann-Whitney *U* test. We performed Student’s *t*-tests to compare [oxy-Hb] changes between the two groups. For this purpose, we calculated grand average waveforms every 0.1 s in each channel. This analysis enabled a more detailed comparison of [oxy-Hb] changes along the time course of the task. Data analyses were conducted using MATLAB 6.5.2 (Mathworks, Natick, MA, USA). We used OT-A4 version 1.63 K (Hitachi Medical Corporation, Tokyo, Japan) to create the overlap display of the grand average waveforms for both groups in Fig 2, and to calculate the mean [oxy-Hb] measurements. As we conducted 24 paired *t* tests, we corrected for multiple comparisons using the false discovery rate (FDR) (two-tailed; we set the value of *q* specifying the maximum FDR to 0.05, so that on average, there would be no more than 5% false positives [56]). The correlation between clinical variables, and between the BIS-11 scores and the mean [oxy-Hb] changes were assessed by Spearman’s rank correlation because of their nonparametric distributions. We used PASW Statistics 18.0 J for Windows (SPSS, Tokyo, Japan) for the statistical analyses.

## Results

### Demographic and clinical data

The demographic characteristics of the study participants are presented in Table 1. The participant groups did not differ in terms of mean age, sex, or JART IQ (*t*<0.56, *p*>0.21 for all 3 variables). The results of the psychological examinations are shown in Table 2. The mean PANSS subscale scores for the MAP group were 15.86 (SD, 4.05) for the positive subscale, 16.79 (SD, 7.02) for the negative subscale, and 35.21 (SD, 9.16) for general psychopathology. Importantly, the SCWC-1 (*t* = −2.13, *df* = 33, *p*<0.05,), SCWC-2 (*t* = −3.14, *df* = 33, *p*<0.01), and SCWC-3 (*t* = −3.50, *df* = 33, *p*<0.001) scores were significantly lower in the MAP group relative to the controls. Furthermore, patients with MAP exhibited significantly higher scores on the total (*t* = -5.18, *df* = 33, *p*<0.001) and subscale (attention; *t* = -2.55, *df* = 33, *p*<0.01, motor; *t* = -4.09, *df* = 33, *p*<0.001, non-planning; *t* = -5.69, *df* = 33, *p*<0.001) BIS-11 scores compared with control participants, suggesting that patients with MAP have higher levels of impulsivity.

**Table 2:** Participant symptom scores.

Variable	Patients with MAP		Controls		*p* value
	(n = 14)		(n = 21)		
	Mean	SD	Mean	SD	
**PANSS subscales**					
Positive	15.86	4.05	NA		NA
Negative	16.79	7.02	NA		NA
General psychopathology	35.21	9.16	NA		NA
**BIS-11**					
BIS-11 total score	78.00	5.53	61.55	10.22	<0.001
BIS-11 attention	20.14	3.53	15.95	4.06	<0.01
BIS-11 motor	24.21	4.39	18.75	3.45	<0.001
BIS-11 non-planning	34.86	2.82	26.85	4.45	<0.001
**SCWC**					
SCWC-1	35.93	12.06	44.71	9.25	<0.05
SCWC-2	38.00	12.75	50.43	8.82	<0.01
SCWC-3	37.21	15.12	52.00	8.06	<0.001

MAP, methamphetamine-induced psychosis; SD, standard deviation; PANSS, Positive and Negative Symptoms Scale; BIS-11, The Barratt Impulsiveness Scale Version 11; SCWC-1, SCWC-2, SCWC-3; number of correct answers during the first, second, and third Stroop color-word task trials, respectively; NA, not applicable

All, *t*-test were used.

### Correlation between Stroop color-word task performance and clinical features of MAP

Because SCWC scores on the Stroop-word color task varied across the patients with MAP and the controls, we calculated Spearman’s rank correlations between SCWC scores, age, JART-IQ score, and BIS-11 scores for all participants (Table 3). In the MAP patient group, the SCWC-1 and SCWC-3 scores were positively correlated with JART-IQ (*ρ* = 0.782, *p*<0.01 and *ρ* = 0.648 *p*<0.05), while none of SCWC scores were significantly correlated with age (*ρ*<0.532, *p*>0.21 for all 3 variables). In the control group, the SCWC-2 and SCWC-3 scores were positively correlated with JART-IQ (*ρ* = 0.575, *p*<0.01 and *ρ* = 0.519, *p*<0.05), whereas none of SCWC scores were significantly correlated with age (*ρ*<0.398, *p*>0.15 for all 3 variables). The total BIS11 score was negatively correlated with the SCWC-1 score in MAP group only (*ρ* = −0.619, *p*<0.05).

**Table 3:** Correlations between Stroop task performance and participant characteristics.

	Patients with MAP			Controls		
	(n = 14)			(n = 21)		
	SCWC-1	SCWC-2	SCWC-3	SCWC-1	SCWC-2	SCWC-3
Age	0.326	0.353	0.334	−0.247	−0.398	−0.307
JART-IQ	0.782**	0.532	0.648*	0.259	0.575**	0.519*
BIS-11 total score	−0.619*	−0.373	−0.478	-0.001	-0.168	0.277

MAP, methamphetamine-induced psychosis; JART, Japanese Adult Rating Test; SCWC-1, SCWC-2, SCWC-3; number of correct answers during the first, second, and third Stroop color-word task trials; BIS-11, The Barratt Impulsiveness Scale Version 11.

* *p*<0.05

**p<0.01

All, Spearman’s rank correlations and a multiple comparison test with a false discovery rate (FDR) correction were used.

### The mean percentage of errors during the Stroop color-word task

**Fig 3:**
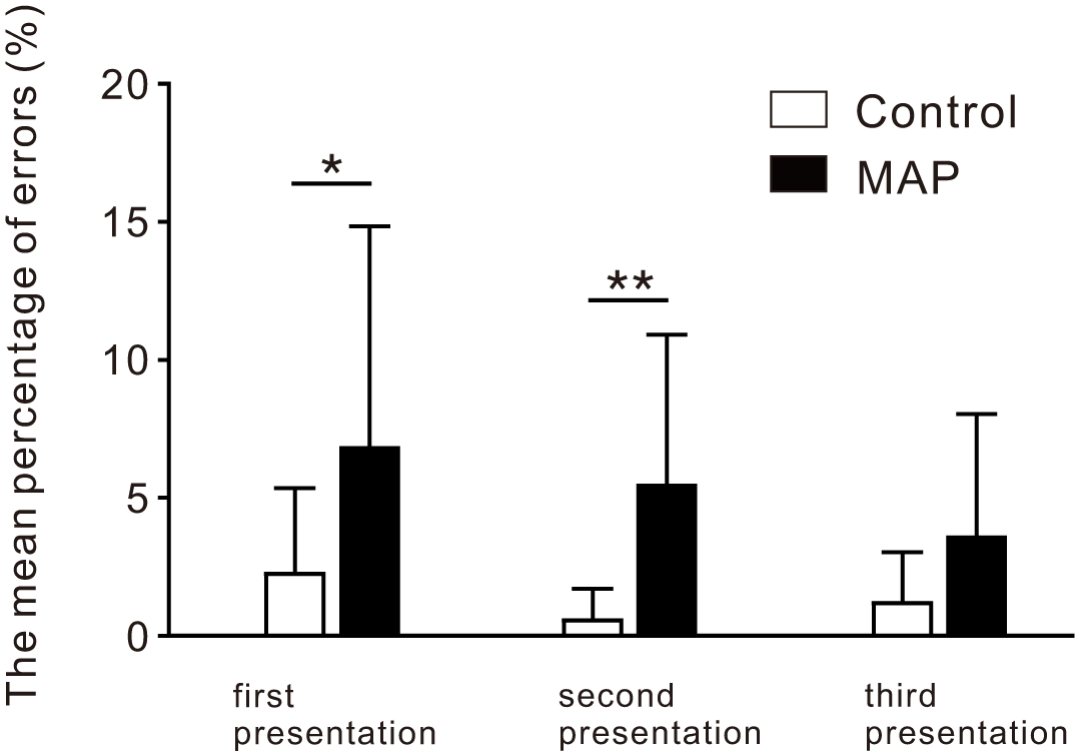
Mean percentage of errors during the incongruent Stroop color-word task. Comparison of mean percentage of errors between methamphetamine induced psychosis (MAP) and control groups during the incongruent Stroop color-word task. The black and open bars represent the MAP group and controls, respectively. *p<0.05, **p<0.01. Abbreviations: MAP, methamphetamine-induced psychosis.

To examine whether disrupted inhibitory control in individuals with MAP was associated with the frequency of incorrect responses given during trials, we assessed the mean percentage of errors made during the incongruent Stroop color-word task (Fig 3). We found that patients with MAP exhibited a higher mean percentage of errors on the first presentation (*U* = 87.50, *p*<0.05) compared with controls. Similarity, we observed a higher mean percentage of errors on the second presentation (*U* = 53.50, *p*<0.01) in patients with MAP relative to controls.

### Hemodynamic responses while performing the Stroop color-word task as measured by NIRS

**Fig 4:**
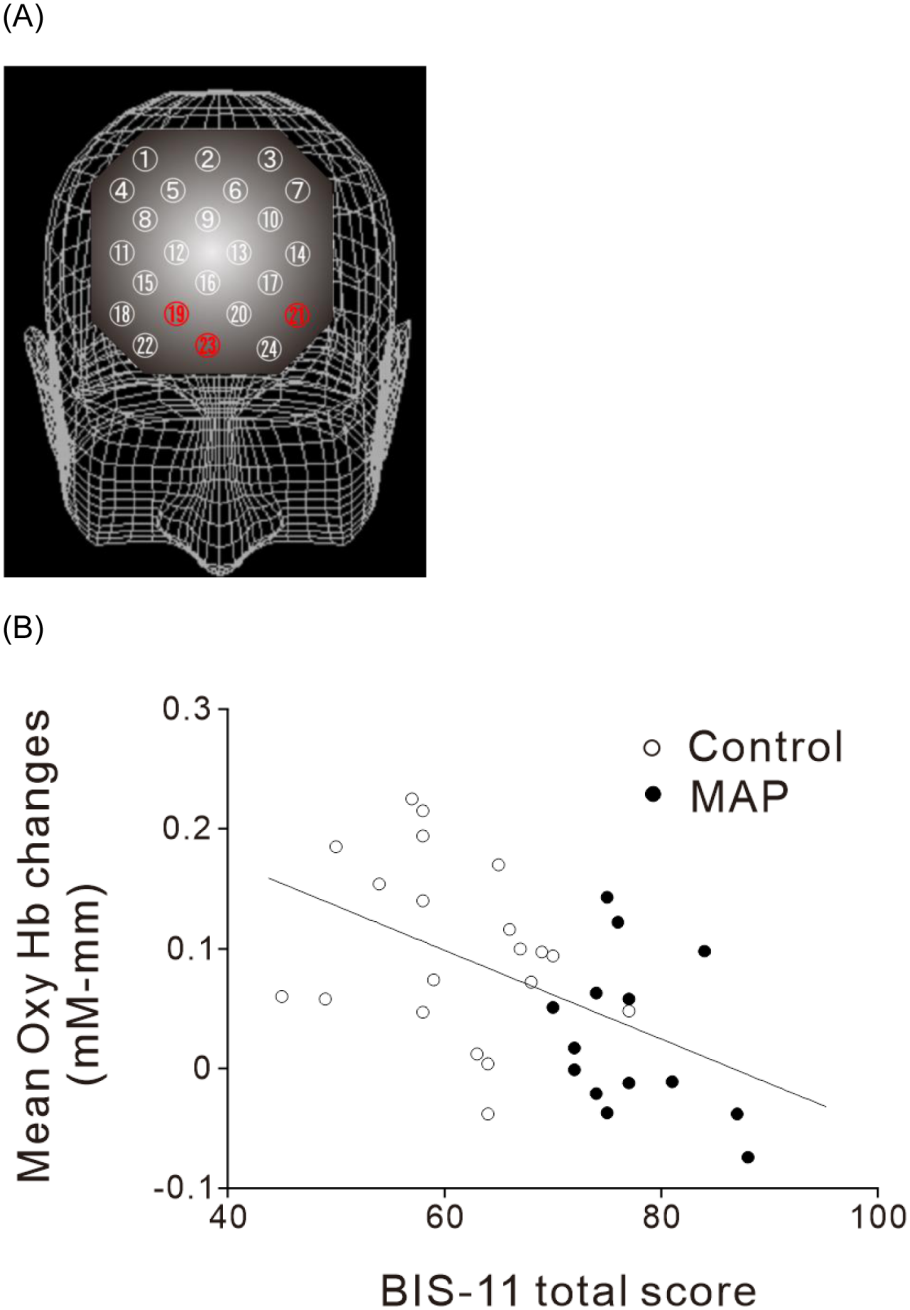
Correlation between total BIS-11 score and hemodynamic change in all participants. (A) Three-dimensional topographical mapping of the channels showing significant correlations between total BIS-11 score and hemodynamic response (Ch 19, 21, and 23, labeled in red). (B) A representative scatter plot showing significant correlation in Ch 19 (*ρ* = −0.537, *p*<0.01). The black and open circles represent the MAP group and controls, respectively. Abbreviations: Ch, channel; BIS-11, Barrett Impulsivity Scale-11; Hb, hemoglobin.

The grand average waveforms of [oxy-Hb] changes during the Stroop color-word task in the two participant groups are illustrated in Fig 2. The grand average waveforms of [oxy-Hb] change appeared to increase substantially during task performance in the control participants while only a small increase was observed in the MAP group. We found significant group differences in the mean [oxy-Hb] measurements between baseline and activation tasks. Of the 24 channels, the mean change in [oxy-Hb] was significantly smaller in channels 19, 21, and 23 in the MAP group relative to the control group (Figs 2 and 4a; FDR-corrected, all *p*<0.01). Indeed, these channels were nearly identical with the FPPFC and APFC (BA 10). Finally, to examine if the hemodynamic changes in these locations were associated with impulsivity, we conducted Spearman’s rank correlation analyses between the hemodynamic changes and impulsivity scores from the total BIS-11 score. We found that the total BIS-11 scores were negatively correlated with the hemodynamic changes in channel 19 (*ρ* = −0.537, *p*<0.01), channel 21 (*ρ* = −0.352, *p*<0.05), and channel 23 (*ρ* = −0.529, *p*<0.01) in all participants (Fig 4b). Of the three channels, the hemodynamic changes in channel 19 were significantly correlated with the total BIS-11 score in patients with MAP (*ρ* = -0.619, *p*<0.01), but not in controls (*ρ* = -0.256, *p* = 0.48), when considered separately. However, the correlations between them in channel 21 and 23 failed to reach the significant level within patient with MAP or controls, probably due to the smaller range of values within each subject group.

## Discussion

To the best of our knowledge, this is the first NIRS study to compare the prefrontal hemodynamic response during the Stroop color-word task in individuals with MAP and controls. We found that the MAP group showed higher levels of impulsivity, as measured by the total and subscale scores of BIS-11, compared with controls. Additionally, those in the MAP group had lower SCWC scores and a higher mean percentage of errors on the Stroop color-word task, which is at least in part due to deficits in inhibitory control. Finally, our NIRS measurement revealed that, compared with controls, those in the MAP group showed significantly smaller [oxy-Hb] changes in channels 19, 21, and 23 in the PFC, during the Stroop color-word task. These data suggest that patients with MAP have significantly lower [oxy-Hb] changes patterns of blood oxygenation in the FPPFC and APFC (BA 10) compared with controls, which appears to be associated with higher impulsivity in these subjects.

Neuropsychological studies have reported that active MA dependent individuals exhibit impaired memory, abnormal manipulation of information, and atypical response inhibition with respect to habitual prepotent responses [57, 58]. As mentioned above, MA-dependent individuals exhibit an abnormal task-relevant response during the Stroop color-word task [57]. Similar findings have been observed in MA dependent participants after a short period of abstinence [14, 15, 59]. In the present study, we found that individuals with MAP had both lower SCWC scores and a higher mean percentage of errors on the Stroop color-word task. We found positive correlations between SCWC scores and premorbid IQ, whereas SCWCs were not correlated with age in either group. Since the Stroop color-word task performance represented as SCWC scores have been linked with impulsivity in other psychiatric disorders [31, 48], we suggest that the Stroop color-word task might be also useful for estimating the severity of impulsivity in individuals with MAP. Given the association between PFC activity and inhibitory control relevant to addiction [60–63], abnormal performance on the Stroop color-word task in the MAP group might be associated with reduced PFC activity as measured by NIRS.

Several researchers have used neuroimaging techniques, including NIRS, to investigate the characteristics of MAP. Consistent with the present study, Okada et al. used NIRS to show that patients with MAP exhibited significantly smaller changes in [oxy-Hb] at the FPPFC during stop-signal tasks which is another task for the assessment of inhibitory control. These individuals also had higher impulsivity compared with controls and schizophrenia patients when assessed by PANSS excitement score [32]. These findings are consistent with the notion that the FPPFC and its junction with the temporal lobe are the neuronal basis of impulsivity and moral judgment [64–66]. In addition, Salo et al. used fMRI to demonstrate that MA dependent participants displayed reduced activation in the right PFC compared with control participants when evaluating conflict [26]. Moreover, previous investigations have reported hypofunction in the anterior cingulate cortex during go/no-go task performance in MA dependent individuals [24]. In the present study, the Stroop color-word task was used as stimulation because previous studies revealed the association between prefrontal hemodynamic responses and the Stroop color-word task performance using NIRS in healthy controls and/or subjects with other psychiatric disorders [31, 67–70]. We found that patients with MAP exhibited significantly lower changes in [oxy-Hb] during the Stroop color-word task compared with controls, which was relevant to higher impulsivity measured by the BIS-11. This abnormality was localized to the FPPFC and APFC, where moral judgment and impulsivity are thought to be controlled and managed [71, 72]. Taken together, our findings suggest that hypofunction in these regions might lead to deficits in inhibitory control in individuals with MAP. However, there was no significant correlation between the prefrontal hemodynamic responses and the SCWC scores. Given that other domains such as cognitive control and selective attention are also responsible for the performance of incongruent trial on the Stroop color-word task [73–75], future studies linking reduced prefrontal hemodynamic response specifically to impulsivity in patients with MAP are needed to validate this hypothesis.

The present study has several potential limitations. First, the spatial resolution for the detection of hemodynamic responses from the scalp surface using NIRS is lower than that of fMRI, single-photon emission computed tomography (SPECT), or positron emission tomography (PET). However, the spatial resolution may be within an acceptable range because previous NIRS studies have also found clear distinctions in hemodynamic responses between diagnostic groups [31, 47, 48, 76]. Second, all of MAP participants were receiving antipsychotic, anticholinergics and/or anxiolytics during the experiment. Therefore, we could not rule out the potential effects of prescribed drugs on the measurements during the employed task performance. However, previous studies suggested that prefrontal activation was not significantly affected by antipsychotics [77] and that atypical antipsychotics were likely to be beneficial to cognition [78]. In addition, the dosage of each medication was not correlated with the mean changes in [oxy-Hb] in patients with MAP, respectively (all *p*>0.05). Nonetheless, investigating those who are “drug-naïve” or “drug-free” should be considered. Third, our sample size was relatively small. Taken together, future studies using larger samples are required to determine whether reduced hemodynamic change is a conserved feature in individuals with MAP, and to examine whether such changes measured by NIRS are dose-dependent in patients with MAP.

## Conclusion

We have shown that patients with MAP performed poorly on the Stroop color-word task, with an associated reduction in prefrontal hemodynamic responses, compared with controls. Additionally, we found that the degree of hemodynamic activity observed during the Stroop color-word task was negatively correlated with impulsivity. Especially given the quick speed of non-invasive NIRS (about 5 min) compared with other functional imaging methods, the present study suggests that NIRS is easily applicable for identifying the reduced PFC activity associated with impaired inhibitory controls commonly observed in individuals with MAP.

## Abbreviations

MA: methamphetamine
MAP: methamphetamine-induced psychosis
fMRI: functional magnetic resonance imaging
PFC: prefrontal cortex
NIRS: near-infrared spectroscopy
oxy-HB: oxygenated hemoglobin
deoxy-Hb: deoxygenated hemoglobin
BIS-11: Barrett Impulsivity Scale-11
SCID-NP: Structured Clinical Interview for DSM-IV Axis Disorders Non-Patient Edition
JART: Japanese version of the National Adult Reading Test
PANSS: Positive and Negative Symptom Scale
SCWC-1: Stroop color-word task number of correct answers for the first presentation
SCWC-2: Stroop color-word task number of correct answers for the second presentation
SCWC-3: Stroop color-word task number of correct answers for the third presentation
FPPFC: frontopolar prefrontal cortex
APFC: anterior prefrontal cortex
BA: Brodmann area
DLPFC: dorsolateral prefrontal cortex
VLPFC: ventrolateral prefrontal cortex
FDR: false discovery rate
SPECT: single-photon emission computed tomography
PET: positron emission tomography
